# Supplementary material for: Optimization of Canolol Production from Canola Meal Using Microwave Digestion as a Pre-Treatment Method
Source: Foods. 2023 Jan 9;12(2):318. doi: 10.3390/foods12020318 (PMC9857780; doi:10.3390/foods12020318)
Supplement: Supplementary file 1 [file foods-12-00318-s001.zip › foods-2072868-supplementary.pdf]

## Supplementary Data

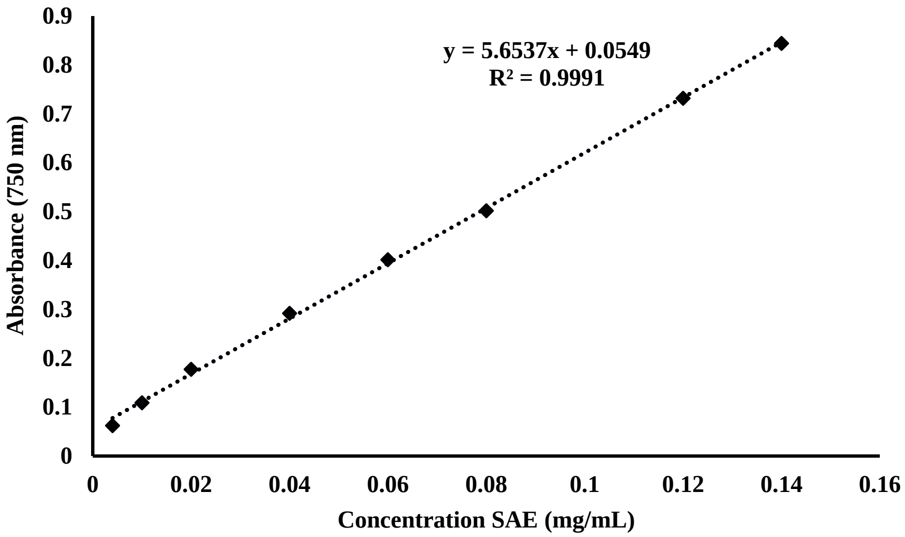


**A**

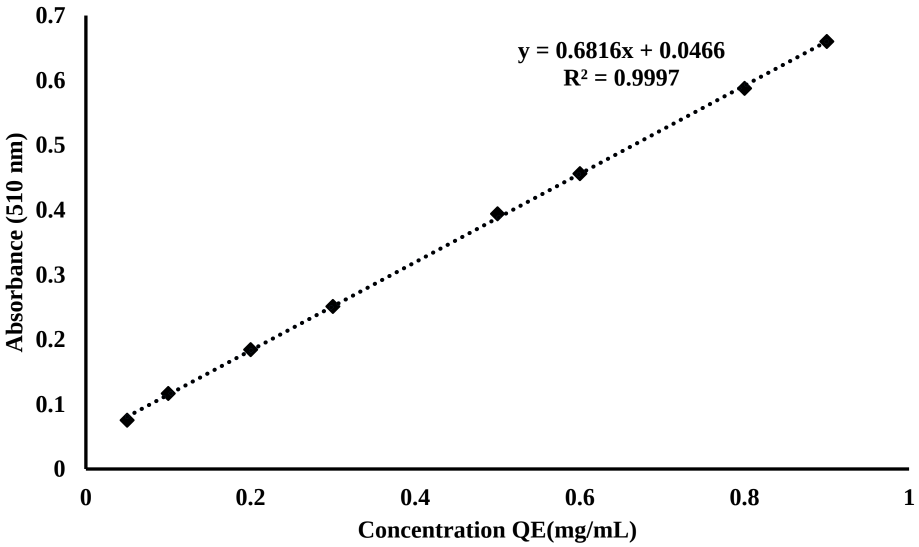


**B**

**Figure S1.** (**A**) Total Phenolic Content Standard Curve using Sinapic Acid Solution (1mM) as Standard (SAE - sinapic acid equivalents, mg - miligram, mL - mililiter, nm – nanometer, $R^2$ - coefficient of variance); (**B**) Total Flavonoid Content Standard Curve

using Quercetin solution (1mM) as  Standard (QE - qercetin equivalents, nm - nanometer, mg - miligram, mL - mililiter, $R^2$ - coefficient of variance).

## Supplementary Data

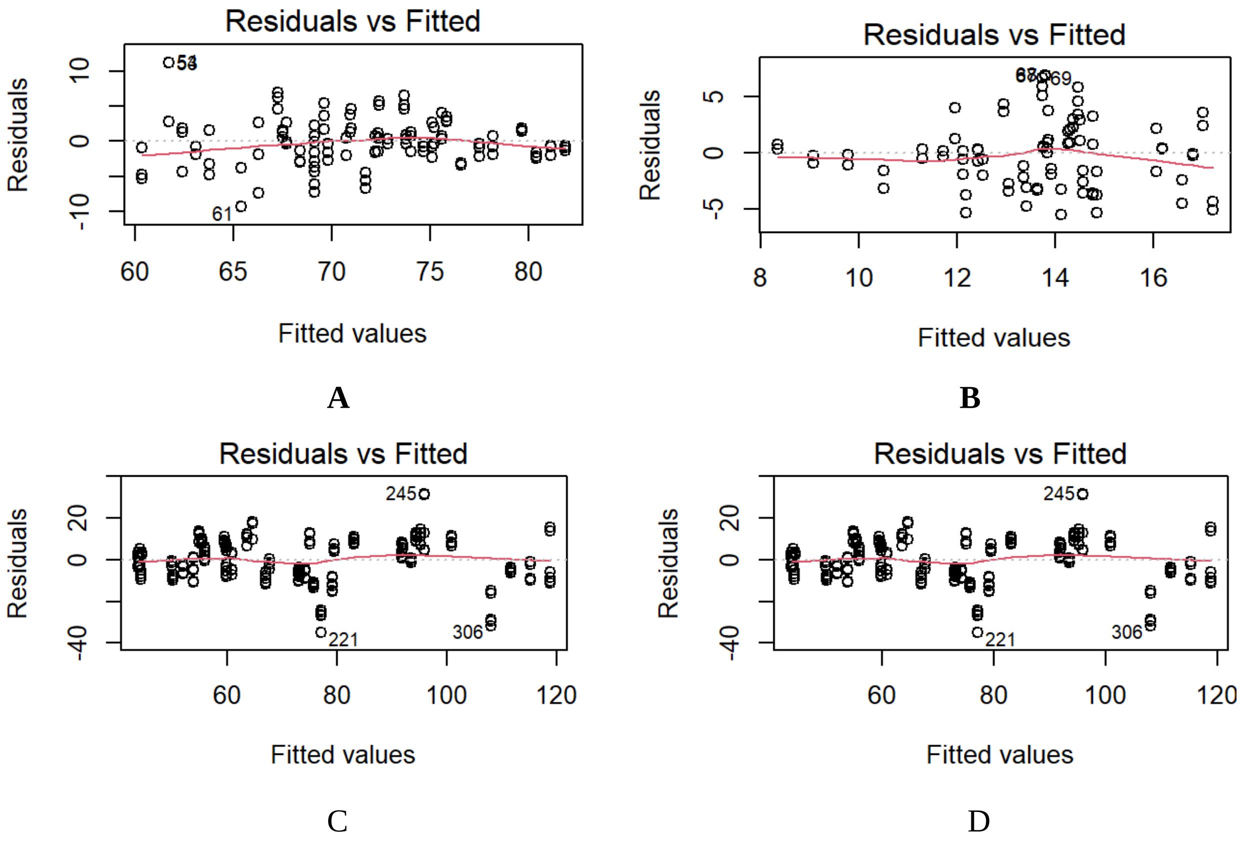


**Figure S2.** Residual Plots for Antioxidant activity (Residuals Vs Fitted Plots) **A** - DPPH activity, **B** - Metal Ion Chelation activity, **C** - Total phenolic content (TPC), **D** - Total flavonoids content (TFC)

## Supplementary Data

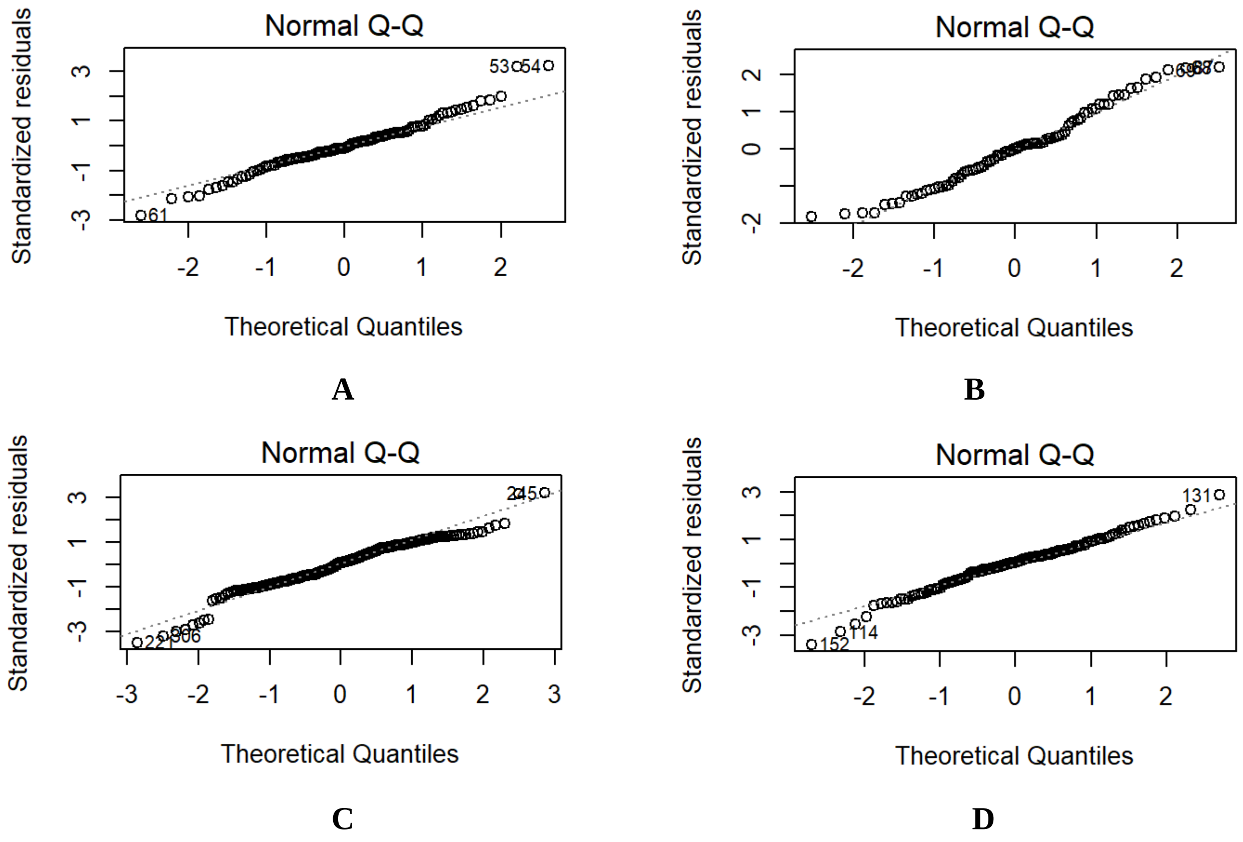


**Figure S3.** Normal Probability Plots for Antioxidant activity A - DPPH activity, B - Metal Ion Chelation activity, C - Total phenolic content (TPC), D - Total flavonoids content (TFC)
